# Compartment-resolved Proteomic Analysis of Mouse Aorta during Atherosclerotic Plaque Formation Reveals Osteoclast-specific Protein Expression*[Fn FN2]

**DOI:** 10.1074/mcp.RA117.000315

**Published:** 2017-12-04

**Authors:** Michael Wierer, Matthias Prestel, Herbert B. Schiller, Guangyao Yan, Christoph Schaab, Sepiede Azghandi, Julia Werner, Thorsten Kessler, Rainer Malik, Marta Murgia, Zouhair Aherrahrou, Heribert Schunkert, Martin Dichgans, Matthias Mann

**Affiliations:** From the ‡Department of Proteomics and Signal Transduction, Max-Planck Institute of Biochemistry, Martinsried, Germany;; §Institute for Stroke and Dementia Research, Klinikum der Universität München, München, Germany;; ¶Comprehensive Pneumology Center, Helmholtz Zentrum München, Member of the German Center for Lung Research (DZL), Munich, Germany;; ‖Klinik für Herz- und Kreislauferkrankungen, Deutsches Herzzentrum München, Technische Universität München, Munich, Germany;; **Department of Biomedical Sciences, University of Padova, Padua, Italy;; ‡‡Institut für Integrative und Experimentelle Genomik, Universität zu Lübeck, Lübeck, Germany;; §§Deutsches Zentrum für Herz-Kreislauf-Forschung (DZHK), e.V., Partner Site Hamburg/Kiel/Lübeck, Lübeck Germany;; ¶¶DZHK e.V. (German Center for Cardiovascular Research), Partner Site Munich Heart Alliance, Munich, Germany

## Abstract

Atherosclerosis leads to vascular lesions that involve major rearrangements of the vascular proteome, especially of the extracellular matrix (ECM). Using single aortas from ApoE knock out mice, we quantified formation of plaques by single-run, high-resolution mass spectrometry (MS)-based proteomics. To probe localization on a proteome-wide scale we employed quantitative detergent solubility profiling. This compartment- and time-resolved resource of atherogenesis comprised 5117 proteins, 182 of which changed their expression status in response to vessel maturation and atherosclerotic plaque development. In the insoluble ECM proteome, 65 proteins significantly changed, including relevant collagens, matrix metalloproteinases and macrophage derived proteins. Among novel factors in atherosclerosis, we identified matrilin-2, the collagen IV crosslinking enzyme peroxidasin as well as the poorly characterized MAM-domain containing 2 (Mamdc2) protein as being up-regulated in the ECM during atherogenesis. Intriguingly, three subunits of the osteoclast specific V-ATPase complex were strongly increased in mature plaques with an enrichment in macrophages thus implying an active de-mineralization function.

Vascular diseases result in almost 18 million fatalities world-wide per year, making them the leading cause of death (1). The underlying pathophysiology is often atherosclerosis and the most common clinical manifestations are stroke and coronary artery disease (CAD)^1^ (2–5). Atherosclerosis is a chronic inflammatory condition characterized by an accumulation of lipids in the arterial wall, together with infiltration of monocytes and other immune cells, leading to the development of an atheromatous plaque. Over time, apoptotic and necrotic cells together with cholesterol crystals form a necrotic core. The surface of advanced plaques is covered by a fibrous cap of variable thickness, composed of a dense collagen-rich extracellular matrix (ECM) and sparsely distributed cells, in particular, vascular smooth muscle cells (VSMCs). In late stages, the expression of proteases can destabilize the cap, inducing plaque rupture and thrombosis, which may manifest as stroke or myocardial infarction (6–11). Thus, plaque stability is directly related to clinical events (12).

The arterial wall structure of vertebrates is subdivided into the tunica intima, media and adventitia, comprising endothelial cells, VSMCs and a heterogeneous population of myofibroblast cells. These cells are embedded in an ECM to provide the biomechanical properties required for vessel function (13–15). The endothelial cells of the tunica intima demark the border to the blood and are crucial in the initial phase of atherogenesis. Systemic risk factors such as hypercholesterolemia, high blood pressure, and disturbed laminar flow induce endothelial dysfunction, subendothelial lipid deposition and leukocyte extravasation (16). Continuous invasion of leukocytes and VSMCs, and the differentiation of macrophages to foam cells elicit cytokine and chemokine secretion, leading to inflammation and to the development of an atherosclerotic plaque. The arterial ECM is critically involved in signaling processes relevant to atherosclerosis and undergoes substantial changes during atherogenesis (17–23). Several studies link ECM components to vascular inflammation and atherosclerotic plaque development (reviewed in (22)), yet our understanding of the underlying protein expression changes is mainly limited to the study of individual factors or small sets of proteins.

Recent application of “omics” methodologies (24), especially of high resolution mass spectrometry (MS), has allowed the categorization of the ECM constituents in “core matrisome” and “matrisome-associated” proteins (25–27). The core matrisome is a multifunctional matrix composed of collagens, proteoglycans and glycoproteins. It provides a biomechanical scaffold, with space-filling and lubrication functions, and plays a role in signal transduction, cell adhesion and many additional physiological processes (28, 29). The matrisome-associated proteins include secreted factors (such as cytokines and chemokines some of which are tightly bound to the ECM), ECM regulators (such as proteinases) and ECM-affiliated proteins (such as galectins) (25).

Although the proteome and secretome in atherosclerosis has been studied by proteomics in mouse models and human samples (30–32), an overall and in-depth picture of the progressive changes during atherogenesis of the protein component of the plaque in general and the ECM is currently lacking (33). MS based analysis of the ECM is challenging because of the insolubility of ECM components as well as contamination from more abundant intracellular proteins (33). Stepwise extraction protocols, in which a biological sample is fractionated according to the solubility of the contained proteins have been described, partially overcoming these limitations (26, 34–37).

To increase our understanding of protein changes in atherogenesis, we sought to study proteome changes with special focus on ECM proteins in an unbiased way. Using advances in MS-based proteomics (38), our laboratory has recently developed a very sensitive and in-depth approach termed quantitative detergent solubility profiling (QDSP) and applied it to unravel matrisome changes during lung injury in great depth (39). Here we applied the QDSP protocol to ApoE knock out mice (ApoE^−/−^), a classical mouse model for atherosclerosis. These mice develop intermediate and advanced plaques at 16 and 24 weeks, respectively, when fed a high-fat, “western-type” diet (40, 41). We generated a quantitative and in-depth resource of time- and compartment-resolved proteome changes of the aorta during atherogenesis. Analysis and replication in an independent cohort retrieved key known factors and uncovered novel proteins pointing to hitherto unknown activities in atherosclerotic plaques.

## EXPERIMENTAL PROCEDURES

### 

#### 

##### Experimental Design and Statistical Rationale

In total, 38 aortas from individual mice were analyzed to generate the data set. For each biological condition (defined by time point, genotype and cohort) we used 3–5 aortas and analyzed them as biological replicates in single injection MS measurements. The control group were age and sex-matched wild type mice (8 weeks, *n* = 5; 16 weeks; *n* = 5; 24 weeks: *n* = 3). Statistical comparisons were performed within cohorts over different biological conditions via ANOVA applying randomization-based FDR control for unequal variance.

##### Animal Experiments

All animal protocols were approved by the government of Upper Bavaria (AZ:175–13) and Schleswig-Holstein (AZ:122–4 (108–9/11)). Mice had *ad libitum* access to food and water and were housed under a 12h light dark cycle. All experiments were done on male littermates (C57BL76J or ApoE^tm1Unc^) fed with standard chow diet. In case of the ApoE^tm1Unc^ mice a high-fat diet (TD88137, Harlan) was administered at 8 weeks age for either 8 or 16 weeks. Animals were sacrificed at age 8, 16 or 24 weeks by an overdose of ketamine and xylazine. The arterial tree was perfused through the left ventricle with 20 ml 0.9% sodium chloride solution. For quantitative detergent solubility profiling (QDSP) aorta were dissected from the ascending part until the bifurcation to the renal arteries and snap frozen in liquid nitrogen and subsequently stored at −80 °C. For histology, the aortic arch and its main branches were dissected and fixed for 24h in 1% PFA solution. The aortic arch was embedded in paraffin and cut into consecutive 4 μm sections using a microtome.

##### Immunohistochemistry

Antibodies against Mamdc2 (HPA021814, Sigma-Aldrich, Taufkirchen, Germany), and Tcirg1 (sc-162300, Santa Cruz Biotechnology, Dallas, TX) were titrated to optimize their working dilution. For subsequent signal amplification the Tyramide signal amplification kit with Cyanine 3 (Cy3) (NEL704A001KT, PerkinElmer, Waltham, MA) was used according to the manufacturer's instruction. To achieve co-staining of macrophages in aortic plaques MAC2 (CL8942AP, Cedarlane, Burlington, Canada) was used together with Alexa 647 conjugated F(ab′)_2_ Fragment (112–606-003, Jackson laboratory, Bar Harbor, ME). Confocal imaging was performed on laser scanning confocal with airyscan (Zeiss LSM 880).

##### Sample Preparation Procedures for Proteome Analysis - QDSP

QDSP of mouse aorta was performed following a previously published protocol with minor modifications (39). Aorta were disrupted in PBS containing 1x protease inhibitor mixture with EDTA (PIC, Roche, Penzberg, Germany) using a micro tissue grinder (Thermo-Fisher Scientific, Waltham, MA). After centrifugation (20 min at 16,000 × *g*) the supernatant was collected and the pellet resuspended in buffer 1 (150 mm NaCl, 50 mm Tris-HCl (pH 7.5), 5% Glycerol, 1% IGEPAL-CA-630 (Sigma-Aldrich), 1 mm MgCl_2_, 1x PIC, 1% Benzonase (Merck, Burlington, MA), 1x PIC). After 20 min incubation on ice, the extract was centrifuged and the supernatant collected and combined with the first supernatant (FR1). The pellet was resuspended in buffer 2 (50 mm Tris-HCl pH 7.5, 5% glycerol, 150 mm NaCl, 1.0% IGEPAL CA-630, 0.5% sodium deoxycholate, 0.1% Sodium Dodecyl Sulfate (SDS), 1% Benzonase (Merck), 1× PIC), incubated on ice for 20min and centrifuged. The supernatant was collected (FR2), the pellet resupended in buffer 3 (50 mm Tris-HCl pH 7.5, 5% glycerol, 500 mm NaCl, 1% IGEPAL CA-630, 2% sodium deoxycholate, 1% SDS, 1% Benzonase and 1× PIC), and incubated at room temperature for 20 min. Following centrifugation, the supernatant was collected (FR3) and the insoluble pellet resuspended in PBS + PIC (INSOL).

All four fractions were acetone precipitated and subjected to in-solution digest as previously described (39, 42). Precipitates were resuspended in denaturation/reduction/alkylation buffer (6 m guanidinium hydrochloride (Sigma-Aldrich), 100 mm Tris-HCl pH 8, 10 mm TCEP (Thermo-Fisher Scientific), 50 mm CAA (Thermo-Fisher Scientific)), incubated at 99 °C for 10 min and sonicated 15 min in a Bioruptor (Diagenode, Liége, Belgium) at high setting with 30 s on 30 s off cycles. Samples from FR1–3 were diluted 10-fold with dilution buffer (10% acetonitrile) and digested over night with Trypsin (Sigma-Aldrich) and LysC (Wako, Neuss, Germany) (1:20 enzyme to protein ratio for each enzyme) at 37 °C. Samples of the INSOL fraction were diluted with 1:1:1 with dilution buffer and U/T buffer (6 m urea, 2 m thiourea) followed by addition of 2.5 μg Lys-C and mechanical disruption applying 200 strokes in a micro tissue grinder. The resulting mixture was sonicated again 15min in the Bioruptor and incubated 3 h at 37 °C under vigorous shaking conditions. The predigested samples were diluted 1:3.3 with dilution buffer and digested over night by addition of 2.5 μg Trypsin and 2.5 μg Lys-C at 37 °C.

Peptides of all four fractions were desalted using stage-tips containing a Poly-styrene-divinylbenzene copolymer modified with sulfonic acid groups (SDB-RPS) material (3M, St. Paul, MN) as previously described (42).

##### LC-MS/MS Analysis

Peptides were separated on a reverse phase column (50 cm length, 75 μm inner diameter) packed in-house with ReproSil-Pur C18-AQ 1.9 μm resin (Dr. Maisch GmbH, Ammerbuch, Germany). Reverse-phase chromatography was performed with an EASY-nLC 1000 ultrahigh pressure system, coupled to a Q-Exactive HF Mass Spectrometer (Thermo Scientific). Peptides were loaded with buffer A (0.1% (v/v) formic acid) and eluted with a nonlinear 120-min gradient of 5–60% buffer B (0.1% (v/v) formic acid, 80% (v/v) acetonitrile) at a flow rate of 250 nl/min. After each gradient, the column was washed with 95% buffer B and re-equilibrated with buffer A. Column temperature was kept at 50 °C by an in-house designed oven with a Peltier element and operational parameters were monitored in real time by the SprayQc software. MS data were acquired with a top15 shotgun proteomics method, where in each cycle a full scan is followed by up to 15 data-dependent MS/MS scans. Target value for the full scan MS spectra was 3 × 10^6^ charges in the 300–1650 *m*/*z* range with a maximum injection time of 20 ms and a resolution of 60,000. Isolation of precursors was performed with the quadrupole at window of 1.4 *m*/*z*. Precursors were fragmented by higher-energy collisional dissociation (HCD) with normalized collision energy (NCE)/stepped NCE of 27. MS/MS scans were acquired at a resolution of 15,000 with an ion target value of 1 × 10^5^, a maximum injection time of 25 ms, and an underfill ratio of 10%. Repeated sequencing of peptides was minimized by a dynamic exclusion time of 20 s.

##### Computational MS-data Analysis

MS raw files were analyzed by the MaxQuant software (43) (version 1.5.3.29) and peak lists were searched against the mouse Uniprot FASTA database (UP000000589_10090.fasta and UP000000589_10090_additional.fasta, version June 2015), and a common contaminants database (247 entries) by the Andromeda search engine. Cysteine carbamidomethylation was set as fixed modification, methionine oxidation and N-terminal protein acetylation as variable modifications. False discovery rate was 1% for proteins and peptides (minimum length of 7 amino acids) and was determined by searching a reverse database. Peptide specificity was set to trypsin requiring C-terminal arginine or lysine with a maximum of two missed cleavages. Maximal allowed precursor mass deviation for peptide identification was 4.5 ppm after time-dependent mass calibration and maximal fragment mass deviation was 20 ppm. “Match between runs” was activated with a retention time alignment window of 20 min, and a match time window of 0.7 min to increase protein identification. For total proteome analyses, solubility fractions were defined in MaxQuant for a combined protein MaxLFQ output, with a minimum ratio setting of 2. For fraction specific analyses no fraction information was predefined and the minimum ratio setting was 1. Protein abundance estimates were calculated by dividing MaxLFQ values by the theoretical number of tryptic peptides per protein.

##### Statistics

All downstream bioinformatic analyses were performed with Perseus (44) (versions 1.5.3.0 and 1.5.5.5) and R (version 3.2.2). Protein groups only identified by site FDR, only from peptides identified also in the reverse database, or belonging to IgG subtypes were excluded from the analyses. For fraction specific analyses, MaxLFQ protein intensity values were filtered for at least two different peptides. For PCA, ANOVA and Student's *t* test, missing values were imputed with a width of 0.2 and a downshift of 1.8 over the total matrix. ANOVA was performed in Perseus with s0 set to 0 and subsequent filtering for a minimum fold change of 1.5-fold. Gene annotation enrichment analyses were done with Fisher's exact test including GOMF, GOCC, GOBP, and Corum annotations, curated gene lists of matrisome (25, 26), immune cell signatures (45, 46), and gene lists taken from Ingenuity Pathway Analysis (IPA, Qiagen, Hilden, Germany). Minimum FDR was set to 0.1 and annotation lists filtered for the presence for at least three proteins in the intersection. Student's *t* test was performed with the SAM package in R (47). The logarithmized MaxLFQ intensities were filtered to have at least two valid values in one of the replicates of a single time point. Missing values were imputed as described before and *t* test statistics calculated using the samr function (s0 = 0.2, nperm = 1000). Delta tables were computed using the samr function with a minimal fold change of 2 and significant outliers defined by the smallest delta value allowing identification of the highest number of significant outliers with a median FDR < 0.1.

## RESULTS

### 

#### 

##### Quantitative Detergent Solubility Profiling (QDSP) of Mouse Aorta

**Fig. 1.**
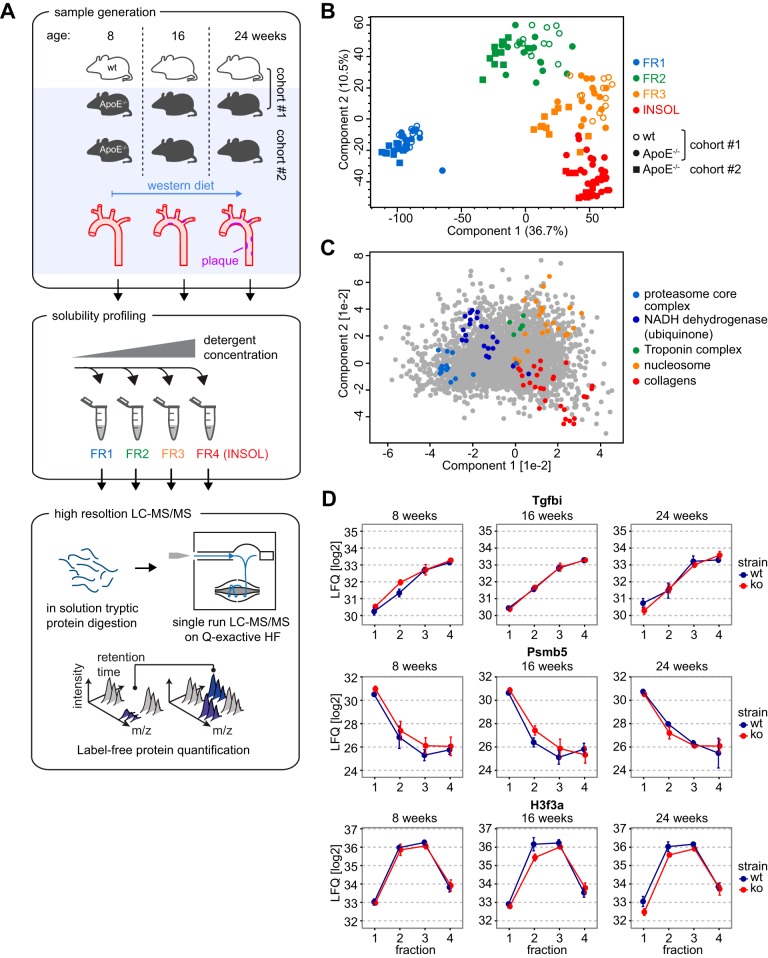
**Quantitative detergent solubility profiling of mouse aorta.** *A*, Outline of the experimental setup. Two independent ApoE^−/−^ mouse cohorts and one paired wild-type mouse cohort were analyzed at the age of 8, 16 and 24 weeks (*n* = 3 - 5 mice per time point). Aortas were prepared and proteins sequentially extracted with buffers of increasing detergent strength. Proteins from the four different fractions were digested with trypsin and analyzed by high-resolution mass spectrometry in a label free shotgun approach. *B*, Principal component analysis(PCA) reveals a clear separation of the four fractions in the first two components. *C*, Protein loadings of the PCA shown in (*B*). *D*, QDSP profiles for selected proteins of cohort #1. Data points are filtered for the presence of at least two valid values and are averages. Error bars represent S.E.

To follow atherosclerotic plaque development by proteomics, we fed 8 week old atherosclerosis prone ApoE^−/−^ mice for 8 and 16 weeks with a high-fat, western-type diet (Material and Methods) (Fig. 1*A*) resulting in consistent formation of atherosclerotic lesions (supplemental Fig. S1*A*). As controls, we used age- and sex-matched wild-type mice fed with chow diet, avoiding spontaneous plaque formation. For an independent evaluation of our data, we included a second ApoE^−/−^ mouse cohort, which was kept in a different mouse facility. Per cohort, each biological condition consisted of three to five individual animals.

To gain insight into the cellular localization of proteins we applied the QDPS protocol (39). Briefly, we lysed individual aortas in buffer with low detergent strength, pelleted homogenized material by centrifugation and performed consecutive extraction steps with buffers containing increasing detergent concentrations. The resulting insoluble fraction was mechanically sheared and all fractions enzymatically digested by trypsin and LysC. Although the first QDPS fractions contained about 50–100 μg, peptide amounts in the more insoluble ones were about hundred-fold lower. For each aorta we quantified 3756–4728 proteins (median 4,265) using label-free values (MaxLFQ) calculated by the MaxQuant software (43, 48) (supplemental Table S1, supplemental Fig. S1*B*). We achieved high technical and biological reproducibility as reflected in Pearson correlation coefficients above 0.90 for all aorta proteomes (supplemental Fig. S1*C*).

Reproducibility was also high at the level of individual fractions as evident from principal component analysis (PCA) (Fig. 1*B*). The four fractions clearly separated in the PCA plot in the first two components, despite being collected from two different and independently processed cohorts. cytosolic proteins such as the proteasome complex core members showed specific enrichment toward the first fraction (FR1), membrane proteins such as the NADH dehydrogenase complex were localized between FR1 and FR2 (Fig. 1*C*). The tropomyosin associated troponin complex specifically purified in FR2, and nucleosomal proteins distributed over FR2 and FR3. As expected, the ECM proteins such as collagens were preferentially enriched toward the insoluble fraction (FR4). The robustness of the protocol at the level of individual protein is exemplified by the highly reproducible profiles of Transforming Growth Factor Beta Induced (Tgfbi), Proteasome Subunit Beta 5 (Psmb5), and Histone H3.3 (H3f3a) across different time points, conditions and cohorts analyzed (Fig. 1*D*, supplemental Fig. S1*D*). This in-depth proteomic data set of mouse aorta during atherogenesis robustly reports on the compartmentalization of proteins within the tissue based on their detergent solubility profiles.

##### Atherogenesis and Vessel Maturation Lead to Distinct Proteome Changes in the Aorta

**Fig. 2.**
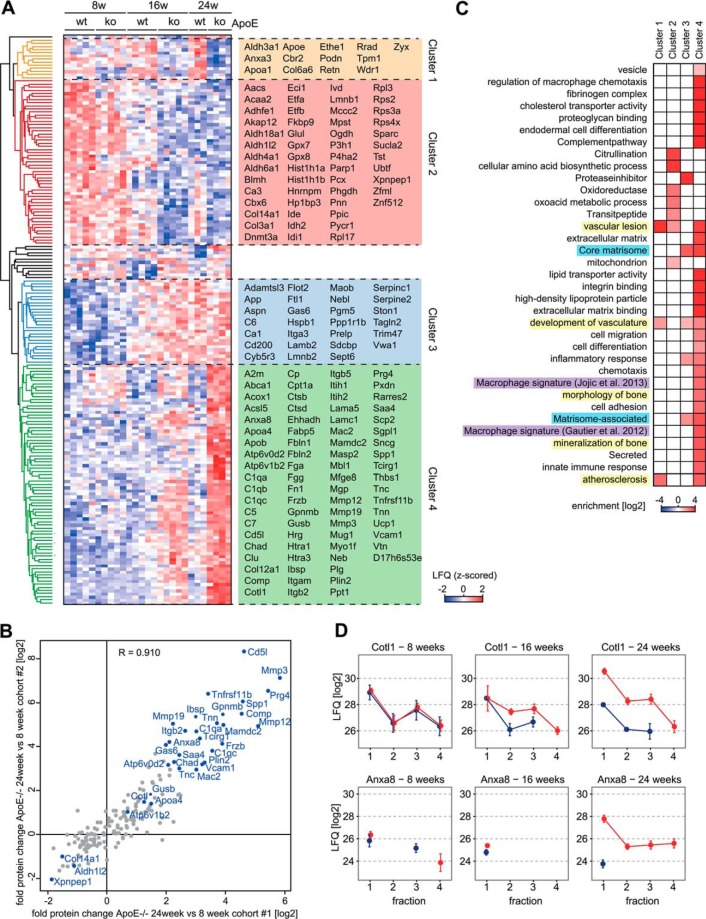
**Total proteome changes during atherogenesis.** *A*, Unsupervised hierarchical clustering of significantly (ANOVA, FDR < 0.05, fold change > 1.5 < −1.5) regulated proteins (z-scored MaxLFQ values) analyzing all fractions of the first cohort together. *B*, Correlation of protein fold changes (ApoE^−/−^, 24 weeks *versus* 8 weeks) of both cohorts reveals low variation. *C*, Gene annotation enrichment analysis of proteins from each cluster *versus* all quantified proteins. In addition to Gene Ontology (GO) annotations (no shading), annotations included extracted gene lists from Ingenuity pathway analysis (IPA) (yellow shading), immune cell gene signatures (Gautier *et al.*, 2012; Jojic *et al.*, 2013) (purple shading), as well as curated lists of matrisomal proteins (Naba *et al.*, 2012a; Naba *et al.*, 2012b) (blue shading) obtained from the literature. *D*, Cotl1 QDPS profile of cohort #1. Data points are filtered for the presence of at least two valid values and are averages. Error bars represent S.E.

The changes in the aortas of our mouse cohorts are because of normal maturation and atherogenesis induced changes in these vessels as well as to plaque formation in the ApoE^−/−^ mice. Note that these plaques constitute only a few percent of aorta mass, requiring accurate and in-depth differential quantification of the proteomes. From our data set, we first calculated protein changes of all four QDPS fractions together, taking advantage of the accurate MaxLFQ normalization implemented in the MaxQuant software (48). Vessel maturation likely occurs to the same extend in both genotypes, whereas atherogenesis-specific changes should only manifest in the ApoE^−/−^ and high-fat fed mice. To define significantly regulated proteins, we therefore performed one-way ANOVA analysis between the genotypes in all of the three time points. Requiring a minimum fold change of 1.5-fold over the average expression level in the biological replicates resulted in 182 significantly regulated proteins in cohort 1 at a False Discovery Rate (FDR) below 0.05 (supplemental Table S2; Fig. 2*A*). Comparing the expression changes of this set of proteins in the independent cohort 2, revealed very high reproducibility of fold changes (*r* = 0.91), clearly validating our findings (Fig. 2*B*).

Unsupervised hierarchical clustering of significantly regulated proteins revealed four major groups (Fig. 2*A*). Although Cluster 2 and 3 contain proteins regulated in response to vessel maturation independently of the genotype, the ones in Cluster 1 and 4 were specifically regulated during atherogenesis in ApoE^−/−^ mice. Cluster 4 contained a large number of known factors related to atherosclerotic plaque development that were up-regulated at the 16 week and particularly the 24 week time points in the knock-out mice. This included the endothelial expressed vascular cell adhesion molecule-1 (Vcam-1) responsible for recruitment of immune cells (49), the macrophage derived Cathepsins B and D (Ctsb/Ctsd) (50), Galectin-3 (Mac2) (51), as well as apoptosis inhibitor of macrophage (AIM/Cd5l) (52), serving as positive controls for atherogenesis mediated recruitment and invasion of monocytes. The proteins with highest regulation in response to the genotype were also robustly enriched in individual *t* test comparisons of ApoE^−/−^ and wild type aortas at the three different time points (supplemental Fig. S2*A*–S2*C*).

Bioinformatic “annotation enrichment analysis” in Cluster 4 revealed a high prevalence of proteins of the matrisome compartment (25, 26), immune cell gene signatures (45, 46) (*p* < 0.01), as well as of the gene lists “atherosclerosis” and “vascular lesion” from Ingenuity pathway analysis (IPA) (*p* < 10^−11^; Fig. 2*C*, supplemental Table S3). Further supporting our analysis, Cluster 4 was specifically enriched for Gene Ontology (GO) annotations related to known molecular processes in atherogenesis, including “chemotaxis” (*p* < 10^−4^), “integrin binding” (*p* < 10^−7^) and “secreted” protein (*p* < 10^−32^). Interestingly, proteins related to “morphology of bone” (*p* < 10^−7^) and “mineralization of bone” (*p* < 10^−3^) were also enriched, pointing to vascular calcification a crucial event in atherogenesis. Our proteomic data directly provides the identity of the proteins responsible for these annotation enrichments (supplemental Table S4).

In addition to a large set of proteins that are biologically linked to atherogenesis, we discovered several factors without previous connection to atherosclerosis. One of these is Coactosin-like protein (Cotl1), a chaperone for the Arachidonate 5-lipoxygenase (Alox5) (Fig. 2*D*). Alox5 catalyzes an initial step in the biosynthesis of leukotrienes, mediators of smooth muscle cell contraction that act as strong activators of inflammation when overexpressed. Polymorphisms in genes involved in the leukotriene pathway have been linked to atherosclerosis in humans and Alox5 itself is a key molecule for atherosclerosis susceptibility in mice (7, 53). As Alox5 stabilization by Cotl1 leads to increased activity because of an inactivation of protein turnover (54), the up-regulation of Cotl1 observed here may contribute to the activation of the leukotriene pathway during atherogenesis. Another up-regulated protein not previously linked to atherogenesis is Annexin A8 (Anxa8) (Fig. 2*D*). Loss of Anxa8 results in a reduction of the leukocyte adhesion factor P-selectin and its cofactor CD36 at the cell surface (55). Conversely, the up-regulation of Anxa8 during atherogenesis observed here may increase leukocyte recruitment to aortic endothelial cells.

We also identified proteins that were specifically downregulated in atherogenic ApoE^−/−^ mice compared with nonatherogenic wild type mice at the age of 16 and 24 weeks (Fig. 2*A*). Podocan (Pdn), is an inhibitor for the migration and proliferation of VMSCs in mice and humans (56). As Pdn^−/−^ mice show increased neointima formation after arterial injury, its down-regulation might be functionally associated with plaque development. Similarly, the GTPase Rrad (Ras associated with diabetes) suppresses the attachment and migration of VSMCs (57).

##### Atherosclerosis in General and the ApoE^−/−^ Phenotype in Particular are Closely Linked to Lipid Metabolism and Transport

In our data set, 25 out of the 90 proteins that were differentially regulated between wild-type and the ApoE^−/−^ mice on the high fat diet were related to lipid metabolism and transport (supplemental Fig. S2*D*). Most of those proteins were up-regulated specifically during atherogenesis - such as Apolipoprotein B (Apob) and Apolipoprotein A-IV (Apoa4). In contrast, Apolipoprotein A-I (Apoa1) was about 2-fold downregulated and we attribute this to the ApoE knock out rather than to atherogenesis because it was the case at all time points.

Interestingly, palmitoyl protein thioesterase 1 (Ppt1) was specifically up-regulated in ApoE^−/−^ mice after 16 weeks, corresponding to 8 weeks of high-fat diet (supplemental Fig. S2*D*). Ppt1 is a lysosomal hydrolase that removes long fatty acid sidechains such as palmitate from proteins. Mutations in the Ppt1 genes cause infantile neuronal ceroid lipofuscinosis (58), a severe neurodegenerative disease characterized by an accumulation of ceroid aggregates in lysosomes and leading to neuronal death. As ceroid formation has been also observed in atherosclerotic plaques (59), the up-regulation of Ppt1 during atherogenesis may represent a regulatory mechanism aimed at the removal of ceroids.

##### The Osteoclastic V-ATPase Complex is Expressed in Macrophages of Mature Atherosclerotic Plaques

**Fig. 3.**
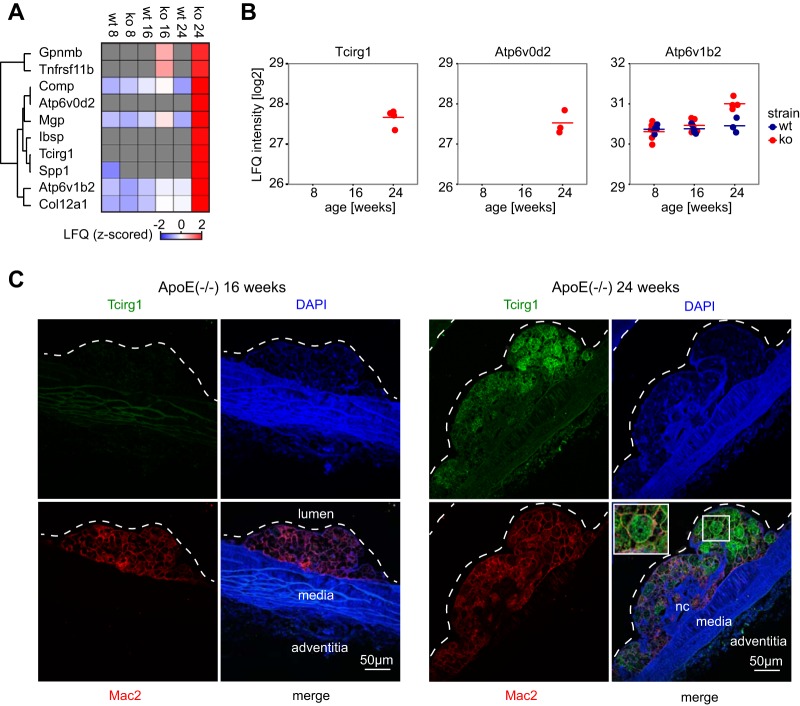
**Regulation of vascular calcification and decalcification pathways.** *A*, Heatmap of mean protein expression (MaxLFQ, z-scored) of significantly regulated proteins with osteoblastic or osteoclastic functions. ko = ApoE^−/−^. *B*, Individual protein expression levels (MaxLFQ values) of three osteoclast specific subunits of osteoclastic V-ATPase complex. *C*, Immunohistochemistry of Tcirg1 reveals staining in MAC2-positive macrophages. Dashed lines delineate the inner vessel wall.

Vascular calcification is a hallmark of atherogenesis, leading to arterial stiffening and mineralization of atherosclerotic plaques (60). Several proteins known to promote calcification were specifically expressed in the ApoE^−/−^ mice during the latest time points of atherogenesis, including Osteoactivin (Gpnmb), Osteopontin (Spp1), Bone sialoprotein 2 (Ibsp), matrix Gla protein (Mgp) and proteoglycan 4 (Prg4) (Fig. 3*A*). Interestingly, two inhibitors of calcification, Osteoprotegerin (Tnfrsf11b) (61) and Cartilage oligomeric matrix protein (Comp) (62) were also increased, suggesting an interplay of positive and negative regulators of vascular calcification.

The mechanisms of vascular calcification resembles the calcification processes in bone (63). Bones are continuously remodeled by decalcifying osteoclast cells derived from the monocyte-macrophage lineage, which balance calcification by the osteoblasts. Aside from secreting enzymes that degrade organic matter, osteoclasts release H+ and Cl- ions to dissolve inorganic matrix by means of a vacular-type H+-ATPase in the plasma membrane, containing three subunits specific to osteoclasts (Tcirg1, Atp6v0d2, and Atp6v1b2). Unexpectedly, we identified all three subunits to be specifically present and up-regulated in atherosclerotic aortas (Fig. 3*B*). Although we detected Tcirg1 and Atp6v0d2 exclusively in 24-week-old ApoE^−/−^ mice, Atp6v1b2 was expressed in all conditions, with a specific increase in 24-week-old ApoE^−/−^ mice. We next asked which cell type expressed this complex. Immunohistochemistry (IHC) against Tcirg1 showed a clear Tcirg1 staining of Mac2-marked macrophages in the plaque, exclusively in 24-week-old mice (Fig. 3*C*). The granular staining further suggested a localization in vesicles in addition to a patchy localization at the plasma membrane. The absence of Tcirg1 in the less developed plaques of 16-week-old ApoE^−/−^ mice establishes a late manifestation of this osteoclastic function during plaque formation, as already suggested by our proteomic results.

##### Matrisome Remodeling During Atherosclerotic Plaque Formation

**Fig. 4.**
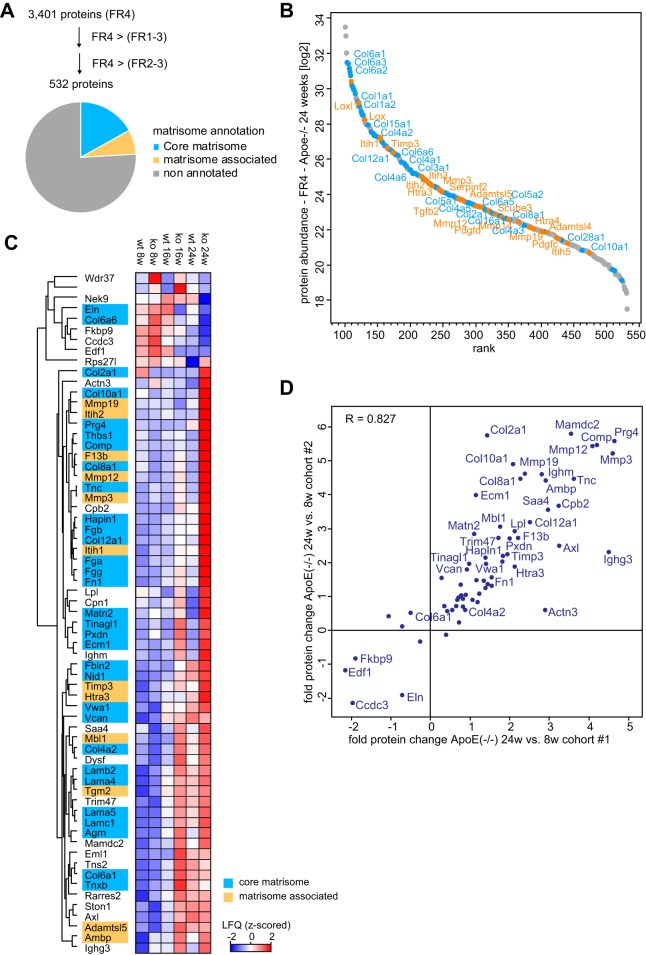
**Matrisome remodeling during atherosclerotic plaque formation.** *A*, Definition of ECM associated proteins based on solubility profiles. *B*, Ranked protein abundance of ECM associated proteins defined in (*A*) in the insoluble fraction of 24 week old ApoE^−/−^ mice. Pale blue: core matrisome proteins, orange: matrisome-associated proteins. *C*, Hierarchical clustering of protein expression values (MaxLFQ, z-scored) in the insoluble fraction of significantly (FDR < 0.1, s0 = 1) regulated ECM-associated proteins defined in (*A*). *D*, Correlation of fold expression changes (24 week *versus* 8 week old ApoE^−/−^ mice) between both cohorts of significantly regulated proteins from the insoluble fraction of cohort #1 reveals low variation.

Structural changes in ECM have been implicated in the pathogenesis of atherosclerosis and other cardiovascular diseases (22). Accordingly, we identified matrisomal proteins to be enriched among significantly regulated proteins during atherogenesis (*p* < 10^−16^). To investigate the remodeling of the matrisome in more detail, we first determined matrisome associated proteins in our data set on the basis of their QDSP expression profiles (Fig. 4*A*, Methods). To this end, we filtered all quantified proteins for at least 2-fold higher expression in the insoluble fraction (FR4) compared with the average expression in FR2 and FR3. We also required at least 2-fold higher MS-signals compared with the average of FR1–3 in at least one biological condition, which resulted in a set of 532 proteins of a total of 3401 proteins initially identified in FR4 (supplemental Fig. S3, supplemental Table S5). As expected, there was high enrichment in ECM annotated proteins (*p* < 10^−42^) (supplemental Table S6), with 25% of proteins being previously designated as core matrisome or matrisome associated proteins (25, 26) (Fig. 4*A*). Collagens are the main structural proteins of the ECM and in terms of absolute abundance, Collagen types VI alpha 1–3 (Col6a1–3) were the most highly expressed ones in aorta, exceeding Collagen types I alpha 1 and 2 (Col1a1,2) 4-fold and Collagen XV type alpha 1 about eight times (Fig. 4*B*). Protein-lysine 6-oxidase (Lox) and Lysyl oxidase homolog 1 (Loxl1), with pivotal roles in the generation of covalently cross-linked collagen structures were the most abundant ECM associated enzymes and had levels comparable to these collagens.

To determine proteins within our ECM related group associated with matrisome remodeling, we performed ANOVA on their expression values in the insoluble fraction over the biological conditions. This resulted in 65 significantly regulated matrisome associated proteins in cohort 1 (FDR < 0.1, minimum fold change 1.5), whose regulation was closely reproduced in cohort 2 (*r* = 0.89) (Fig. 4*C*, 4*D*, supplemental Table S7). Among these proteins we observed strong atherogenesis dependent up-regulation of Collagen types II alpha1 (Col2a1) and XII alpha 1 (Col12a1). Collagen types X alpha 1 (Col10a1), and VIII alpha 1 (Col8a1) were exclusively detected in mature atherosclerotic aortas from ApoE^−/−^ mice. This makes them a specific constituent that contributes to the structure of the plaque matrisome (supplemental Fig. S4).

Proteases actively shape the ECM and, matrix metalloproteases (Mmps) target both core ECM and matrisome associated proteins (23). Mmp3, Mmp12, and Mmp19 were increased during atherogenesis (Fig. 4*C*; supplemental Fig. S4). Up-regulation of Mmp19 has not been reported before, whereas this is already known for Mmp3 and Mmp12 and likely derives from macrophage secretion (22). ECM-associated protease inhibitors were also significantly up-regulated in atherogenesis, including the tissue inhibitor of metalloproteinase 3 (Timp3), which was the most highly expressed one, as well as two subunits of the Inter-Alpha-Trypsin Inhibitor, Itih1 and Itih2. Although there is evidence for a protective role of Timp3 in atherosclerosis (64, 65), Itih1 and Itih2 have not yet been associated with plaque development. The fact that Itih1 was expressed in atherosclerotic aorta in very similar amounts to Timp3, may further support a functional role (Fig. 4*B*).

**Fig. 5.**
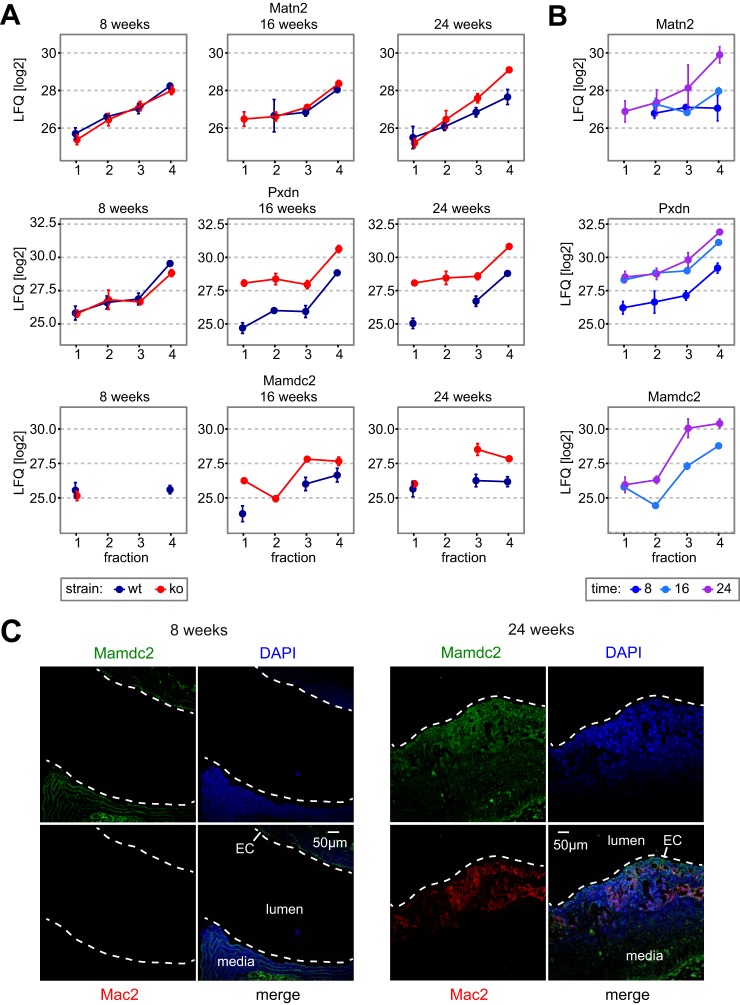
**Identification of novel atherosclerosis-associated ECM proteins.** *A*, *B*, QDSP profiles for ECM-associated proteins with a novel role in atherogenesis in both cohort #1 (*A*) and cohort #2 (*B*). Data points are filtered for the presence of at least two valid values and are averages. Error bars represent S.E. *C*, IHC of Mamdc2 in young healthy (left panel) and mature atherosclerotic aortas (right panel) from ApoE^−/−^ mice. Dashed lines delineate the inner vessel wall (EC: endothelial cell layer).

Apart from proteins connected to known features of plaque matrisomes, our data set contains factors whose involvement in this process was completely unknown. For instance, three matrisomal proteins—Peroxidasin (Pxdn), Matrilin 2 (Matn2), and MAM Domain Containing 2 (Mamdc2), were expressed at low levels in wild-type and young ApoE^−/−^ mice, but were specifically up-regulated in ApoE^−/−^ mice in the course of atherogenesis (Fig. 5*A*, 5*B*). Mamdc2 is an ECM-associated protein of unknown function containing four MAM domains, which can serve as adhesion domains in surface receptors (66). We validated its up-regulation by IHC and found that it was expressed in endothelium and tunica media in non-atherosclerotic aortas and in plaques, concordant with the increase during atherogenesis in the proteomics data (Fig. 5*C*).

## DISCUSSION

Atherosclerosis is a multifactorial disease with a high incidence and mortality, and often clinically manifests as stroke or myocardial infarction (1–5). A classical animal model for atherosclerosis is the ApoE^−/−^ mouse (40, 41). Depletion of the ApoE protein leads to spontaneous hypercholesterolemia and atherogenesis manifested in mature atherosclerotic plaques. Feeding the mice with high fat diet (“Western diet”) accelerates atheroprogression. Molecular events related to vessel maturation happen in conjunction with those related to atherogenesis. To distinguish between them we followed a control cohort (wild-type mice on a chow diet) and contrasted this to an ApoE^−/−^ cohort, fed high fat diet from week eight.

Because of the proteinaceous nature of plaques, it would be most promising to investigate how proteins change in atherogenesis, but so far this has only been done for individual or small sets of candidate proteins. Here, we employed the latest advances in proteomics technology to explore this question. Using aortas from single animals, we obtained quantitative profiles of thousands of proteins across solubility fractions. Although atherosclerotic plaques only constitute a few percent of aorta mass, their proteome was unambiguously captured by comparison to the wild-type mice. Investigation of three time points was crucial, as it provided a proteome base line in wild-type mice as well as early, purely genotype related changes, which we contrasted to both genotype and diet-related changes at the point of fully developed atherosclerosis. Overall, the large majority of atherogenic proteome changes was confined to the 24 week time point—16 weeks after high fat feeding. However, many interesting effects were clearly apparent at 16 weeks already, such as those related to lipid metabolism. Apart from providing a resource of proteome changes in this important disease to the community, our results suggest that many of the established mouse models of human diseases can now fruitfully be interrogated by MS-based proteomics.

The protein expression changes in individual aortas in response to atherogenesis were overwhelmingly upregulations, whereas only few proteins showed decrease in expression. This nonsymmetry can be explained by the *de novo* development of the atherosclerotic plaque, which reflects a buildup of ECM components by VSMCs together with an infiltration of immune cells. The increase in protein expression is therefore the sum of high abundant proteins from immune cells together with the secretion products of all cell types involved in plaque formation.

The quantitative detergent solubility profiling (QDSP) method allowed us to directly measure the compartment association of the aorta proteome. Although previous studies focused on soluble driver proteins in atherogenesis, little is known about changes in the ECM compartment. In addition to the increase in Col2a1, Col10a1, and Col12a1, which are osteoblastic and chondrocytic marker proteins and therefore likely related to plaque calcification (67, 68), we observed an atherogenesis specific up-regulation of Col8a1, a protein that has recently been implicated in fibrous cap formation in atherosclerosis (69). Collagens are organized as highly interlinked networks containing both inter- and intramolecular covalent linkages (29). Peroxidase Pxdn was significantly up-regulated in atherosclerotic aortas both at the total protein level and in the insoluble fraction, which is interesting because it forms collagen IV crosslinked networks in the basement membrane (70, 71). Thus, an increase in Pxdn might contribute to the arterial stiffening that is observed in atherosclerosis (72).

We identified family members of matrix metalloproteinases (Mmps) with both protective and proatherogenic functions to be up-regulated during atherogenesis. Although Mmp3 has been reported to reduce atherosclerotic plaque formation and rupture (73, 74), Mmp12 promotes plaque development and is a risk locus for large artery atherosclerotic stroke (75). Mmp19 was found to be protective against bleomycin induced lung injury in mice (76), therefore its up-regulation during atherogenesis raises the possibility that it has an atheroprotective effect. In addition, we found the Mmp inhibitor Timp3 to be enriched in the insoluble fraction, which has been reported to protect against atherosclerosis (65).

Among the ECM associated and up-regulated proteins that have not yet been related to atherogenesis are Matn2 and Mamdc2. Matn2 is a filament-forming protein widely distributed in ECM of various tissues, with largely uncharacterized function that has been linked to muscle and nerve regeneration as well as inflammation (77, 78). Mamdc2 is a proteoglycan with completely unknown function containing five MAM domains, which mediate interactions and stability and are commonly found in surface receptors (79). Our expression profiles in atherosclerosis make both proteins promising targets for future studies.

Vascular calcification is a crucial hallmark of atherogenic plaques that is causally related to clinical outcomes (60, 63) and here we identified upregulated proteins with known roles in this process. Vascular calcification is an analogous process to bone mineralization, involving osteoblast-like cells that are likely derived from vascular smooth muscle cells (VSMCs) (80). Up-regulated proteins included the bone matrix protein Tnfrfs11b, as well as the cartilage matrix proteins Prg4 and Comp, which serve as intermediates of endochondral bone formation. In addition, Col12a1 and Col10a1, which mark sites of bone and cartilage mineralization, respectively, were enriched in the atherosclerotic ECM. Collectively, these results support an active osteogenesis and chondrogenesis calcification model in atherosclerotic plaque development (63).

In bone, mineralization is a balanced process, which is negatively regulated by osteoclasts. This has led to long-standing speculation that similar osteoclast-like cells might be present in atherogenic plaques and counteract vascular calcification, yet clear evidence for the existence of those cells is missing (63). Here, we unambiguously identified two subunits of the osteoclast specific V-ATPase - Tcirg1 (v0a3) and v0d2-as exclusively expressed in atherosclerotic plaques of 24-week-old ApoE^−/−^ mice. The v1b2 subunit of the same complex, which is also highly expressed in osteoclasts, was likewise increased in atherosclerotic mouse aortas. Notably, we observed Tcirg1 expression in macrophages, thus adding to recent evidence for a role of those cells in mineral clearance within atherosclerotic plaques (81, 82) and suggesting that macrophages adapt an osteoclast-like gene expression program and providing a possible functional mechanism. Osteoclastic V-ATPase is directly involved in bone resorption as it dissolves minerals through continuous expulsion of H^+^ and Cl^−^ ions (83). The osteoclastic V-ATPase might therefore have an important regulatory function against plaque calcification. This finding may have clinical implications, as inhibitors of the osteoclastic V-ATPase have been proposed for the treatment of osteoporosis (83, 84). Because patients with osteoporosis also often develop atherosclerosis (85), inhibiting osteoclastic V-ATPase could unintentionally lead to a detrimental increase in plaque calcification. Although our results derive from an established mouse model, it would be of translational interest to follow them up in the human system. In this case, healthy aorta controls would not be available, but this could be addressed by laser capture based techniques to isolate human plaques specifically.

## DATA AVAILABILITY

The mass spectrometry proteomics data have been deposited to the ProteomeXchange Consortium (http://proteomecentral.proteomexchange.org) via the PRIDE partner repository with the data set identifier PXD006752 (https://www.ebi.ac.uk/pride/archive/projects/PXD006752).

## Supplementary Material

Supplemental Data

## Footnotes

^1^ The abbreviations used are:

CAD: coronary artery disease
ANOVA: analysis of variance
ECM: extracellular matrix
FDR: false discovery rate
MMP: matrix metalloproteinase
MS: mass spectrometry
PCA: principal component analysis
QDSP: quantitative detergent solubility profiling
SEM: standard error of mean
VMSC: vascular smooth muscle cell.
