# Supplementary material for: Compartment-resolved Proteomic Analysis of Mouse Aorta during Atherosclerotic Plaque Formation Reveals Osteoclast-specific Protein Expression
Source: Mol Cell Proteomics. 2017 Dec 4;17(2):321–34. doi: 10.1074/mcp.RA117.000315 (PMC5795394; doi:10.1074/mcp.RA117.000315)
Supplement: Supplemental Data [file supp_17_2_321__index.html]

Compartment-resolved Proteomic Analysis of Mouse Aorta during Atherosclerotic Plaque Formation Reveals Osteoclast-specific Protein Expression — Proteomics of Atherogenesis — Supplemental Data 

# Compartment-resolved Proteomic Analysis of Mouse Aorta during Atherosclerotic Plaque Formation Reveals Osteoclast-specific Protein Expression

## Supplemental Data

- Suppl Figures S1-4 - Suppl Figures S1-4
- Suppl Table 1 - MaxLFQ intensities of all proteins in the dataset
- Suppl Table 2 - Significantly regulated proteins in mouse aorta during atherogenesis
- Suppl Table 3 - Results of the Fisher's excact test on individual clusters of significantly regulated proteins
- Suppl Table 4 - Annotation term associations of significantly regulated proteins
- Suppl Table 5 - ECM-associated proteins in mouse aorta predicted based on QDSP
- Suppl Table 6 - Fisher's exact test on predicted ECM-associated proteins in the mouse aorta
- Suppl Table 7 - Significantly regulated ECM-associated proteins in the insoluble fraction
