# Supplementary material for: Compartment-resolved Proteomic Analysis of Mouse Aorta during Atherosclerotic Plaque Formation Reveals Osteoclast-specific Protein Expression
Source: Mol Cell Proteomics. 2017 Dec 4;17(2):321–34. doi: 10.1074/mcp.RA117.000315 (PMC5795394; doi:10.1074/mcp.RA117.000315)
Supplement: Supplemental Data [file supp_RA117.000315_4948_1_supp_26174_szprv5.pdf]

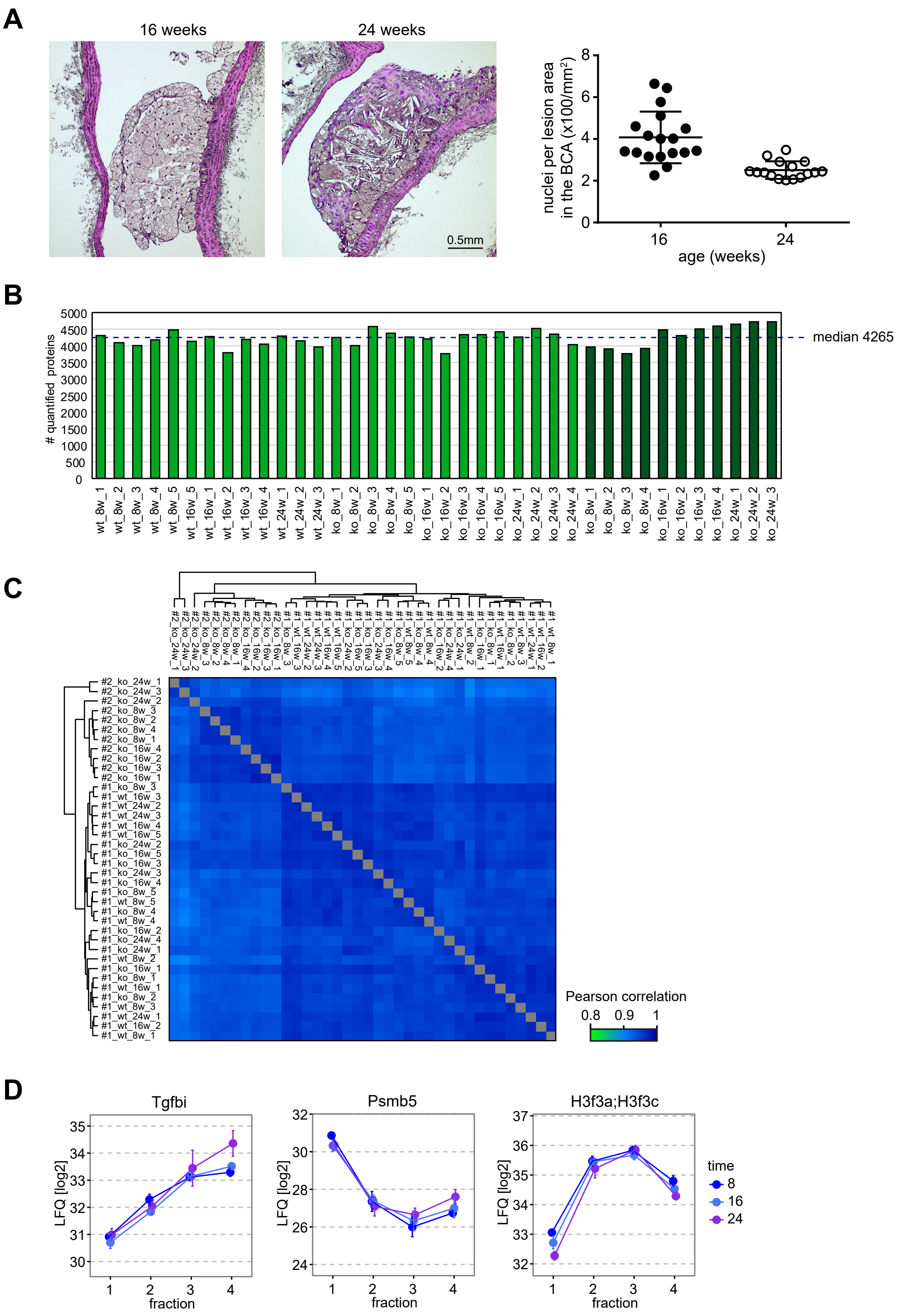

Supplementary Figure S1: Proteomic measurement of mouse aortas with high reproducibility. **(A)** Haematoxylin and Eosin stainings of longitudinal sections of the aorta and nuclei counts per plaque section at Brachiocephalic artery (BCA) lesions (mice n = 2 for each group, 7-9 sections per aorta) revealed different developmental stages of atherosclerotic plaques in 16 week and 24 week old mice **(B)** Number of quantified proteins per mouse aorta by single-run, high-resolution mass spectrometry (MS)-based proteomics. ko = ApoE$^{-/-}$ **(C)** Correlation matrix of full aorta proteomes reveals low variability. **(D)** QDPS profiles for selected proteins in cohort #2. Data points are filtered for the presence of at least two valid values and are averages. Error bars represent SEM.

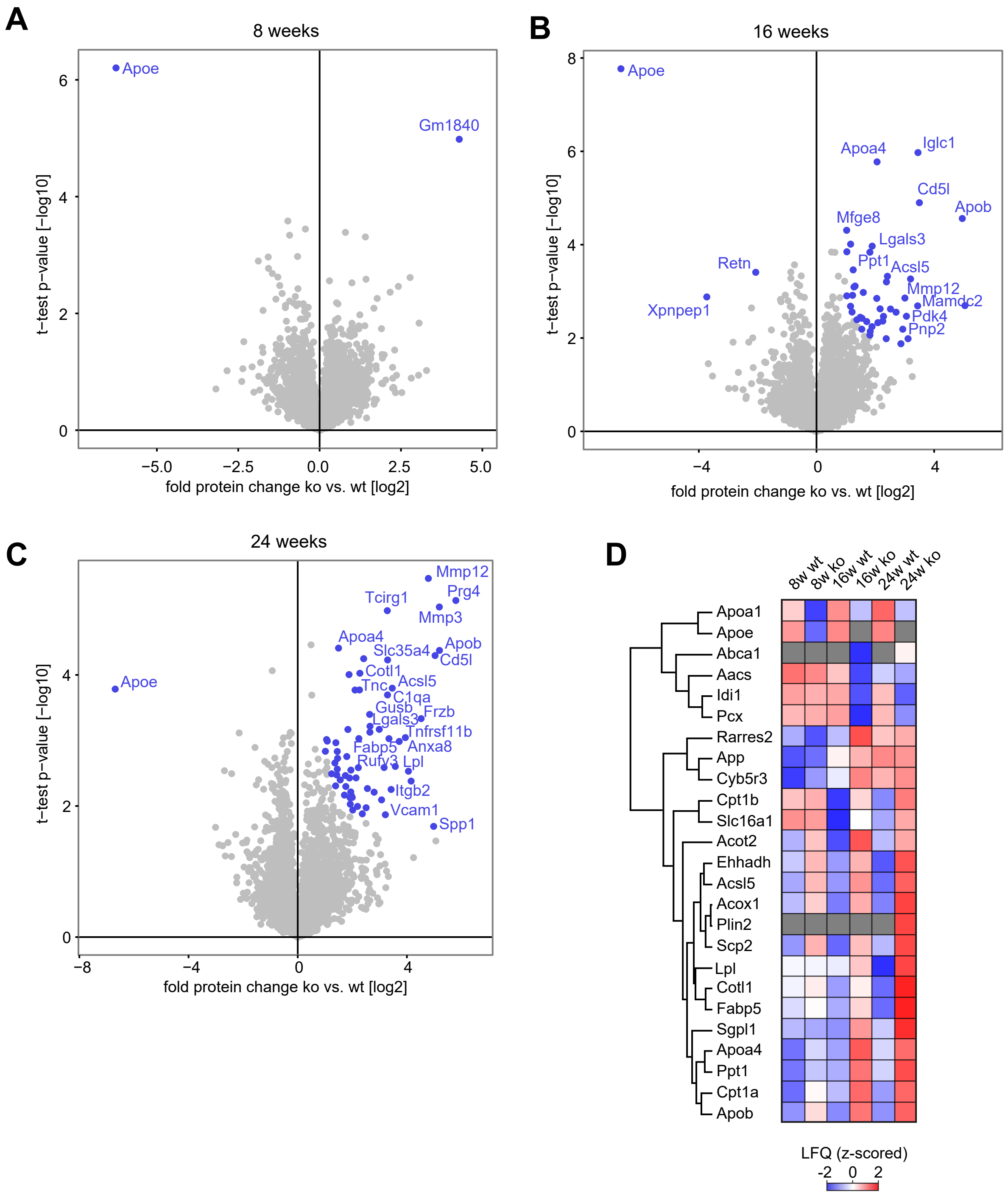

Supplementary Figure S2: (A-C) Student t-test comparisons of ApoE -/- vs. wt mice at different timepoints. Blue proteins: permutation based FDR < 0.1, s0 = 0.2 **(D)** Regulation of proteins related to lipid metabolism and transport in the course of atherogenesis. Unsupervised hierarchical clustering of z-scored MaxLFQ value of significantly (ANOVA, FDR < 0.05) regulated proteins related to lipid metabolism and transport based on GO term annotations and manual database search.

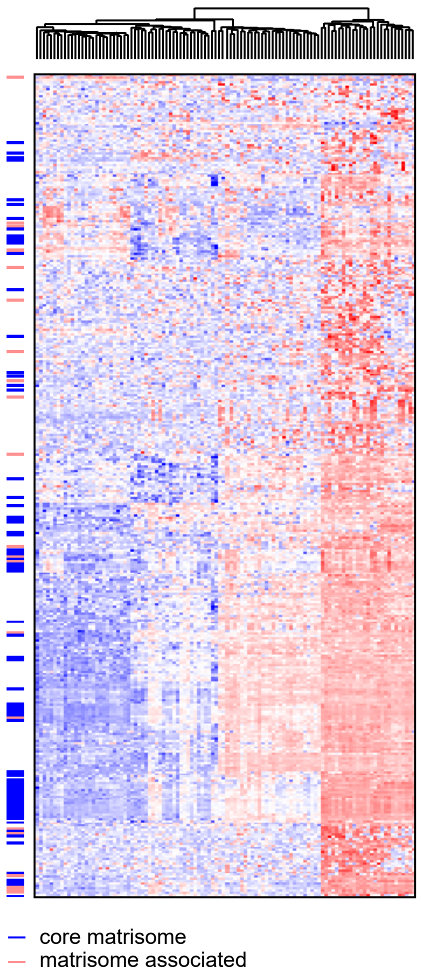

Supplementary Figure S3: ECM associated proteins in mouse aorta. **(**Unsupervised hierarchical clustering of protein expression values (MaxLFQ, z-scored) of ECM-associated proteins as defined by their solubility profiles.

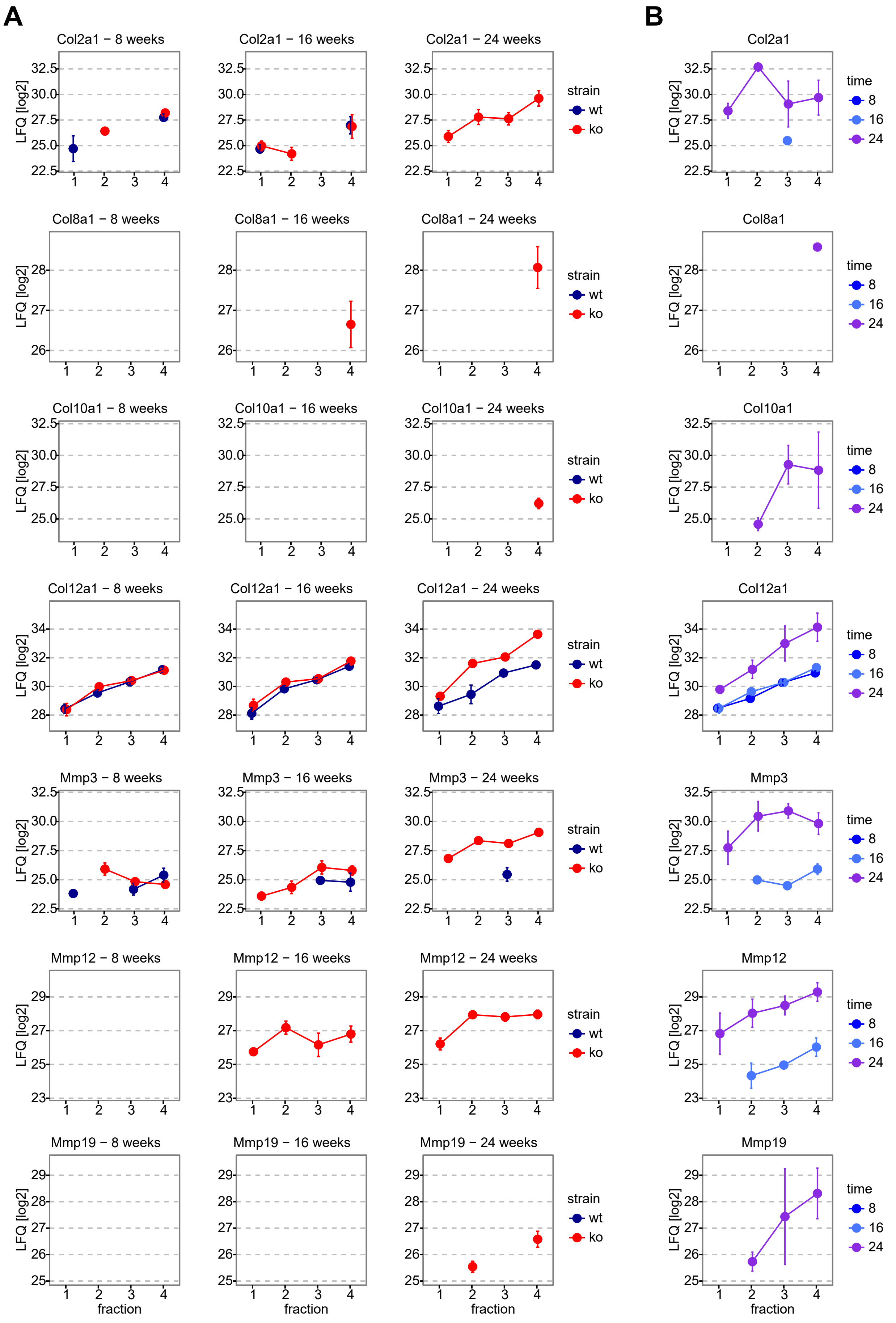

Supplementary Figure S4: Upregulation of collagen subtypes and matrix metalloproteases during atherosclerotic plaque formation. **(A,B)** QDPS profiles of significantly upregulated collagens and MMPs in cohort #1 (A) and cohort #2 (B). Data points are filtered for the presence of at least two valid values and are averages.
